# Understanding critically ill sepsis patients with normal serum lactate levels: results from U.S. and European ICU cohorts

**DOI:** 10.1038/s41598-021-99581-6

**Published:** 2021-10-08

**Authors:** Christopher M. Sauer, Josep Gómez, Manuel Ruiz Botella, David R. Ziehr, William M. Oldham, Giovana Gavidia, Alejandro Rodríguez, Paul Elbers, Armand Girbes, Maria Bodi, Leo Anthony Celi

**Affiliations:** 1 Laboratory for Computational Physiology, Institute for Medical Engineering and Science, Massachusetts Institute of Technology, Cambridge, MA USA; 2 Department of Intensive Care Medicine, Amsterdam Medical Data Science (AMDS), Amsterdam Cardiovascular Science (ACS), Amsterdam Institute for Infection and Immunity (AII), Amsterdam UMC, Vrije Universiteit, Amsterdam, The Netherlands; 3 Department of Intensive Care Medicine, University Hospital of Tarragona Joan XXIII, Dr. Mallafrè Guasch 4, 43005 Tarragona, Spain; 4 Pere Virgili Institute, Tarragona, Spain; 5 Division of Pulmonary and Critical Care Medicine, Department of Medicine, Massachusetts General Hospital, Boston, MA USA; 6 Harvard Medical School, Boston, MA USA; 7 Division of Pulmonary and Critical Care Medicine, Department of Medicine, Brigham and Women’s Hospital, Boston, MA USA; 8 Department of e-Health, Eurecat, Technological Center of Catalonia, Barcelona, Spain; 9 Division of Pulmonary, Critical Care and Sleep Medicine, Beth Israel Deaconess Medical Center, Boston, MA USA; 10 Department of Biostatistics, Harvard T.H. Chan School of Public Health, Boston, MA USA

**Keywords:** Infection, Bacterial infection, Prognostic markers, Fever

## Abstract

While serum lactate level is a predictor of poor clinical outcomes among critically ill patients with sepsis, many have normal serum lactate. A better understanding of this discordance may help differentiate sepsis phenotypes and offer clues to sepsis pathophysiology. Three intensive care unit datasets were utilized. Adult sepsis patients in the highest quartile of illness severity scores were identified. Logistic regression, random forests, and partial least square models were built for each data set. Features differentiating patients with normal/high serum lactate on day 1 were reported. To exclude that differences between the groups were due to potential confounding by pre-resuscitation hyperlactatemia, the analyses were repeated for day 2. Of 4861 patients included, 47% had normal lactate levels. Patients with normal serum lactate levels had lower 28-day mortality rates than those with high lactate levels (17% versus 40%) despite comparable physiologic phenotypes. While performance varied between datasets, logistic regression consistently performed best (area under the receiver operator curve 87–99%). The variables most strongly associated with normal serum lactate were serum bicarbonate, chloride, and pulmonary disease, while serum sodium, AST and liver disease were associated with high serum lactate. Future studies should confirm these findings and establish the underlying pathophysiological mechanisms, thus disentangling association and causation.

## Introduction

Sepsis is a syndrome characterized by “life-threatening organ dysfunction caused by a dysregulated host response to infection”^1^. While sepsis has traditionally been considered the consequence of uncontrolled inflammation, more recent research points to more complex immune-pathogen interactions. While an intact, coordinated immune response is required to clear pathogens, dysregulated activity can result in cell damage, ultimately manifesting as multi-system organ failure^2^.

In the US, approximately half of all admitted patients with sepsis require treatment in the intensive care unit (ICU), contributing to approximately 10–30% of the total ICU population^3–5^. The overall sepsis-associated hospital morality is estimated to be 20–30%^4–7^.

Strenuous physical activity, tissue hypoxia, cellular stress, infection, and various critical illnesses are triggers for accumulation of serum lactate^8^. It is a long-established clinical marker of illness severity^9^. Yet it can also be increased in the absence of cellular hypoxia, whether due to increased glycolysis and increased production, or from decreased clearance^10,11^. Besides these physiological factors, drugs can also increase lactate levels either through induction of glycolysis (e.g., ß2-adrenergic agonists such as albuterol or epinephrine) or inhibition of lactate metabolism (e.g., metformin)^12^.

Since the 1960s, serum lactate has evolved into a well-established marker of illness severity and prognosis, in particular in patients with sepsis^13–15^. Furthermore, the SEPSIS-3 definition of septic shock is based on serum lactate level > 2 mmol/L and hypotension despite adequate volume resuscitation^1^. More recently, Seymour et al. identified sepsis phenotypes and hereby found statistically significant differences in serum lactate levels between the groups^16,17^.

While critically ill patients with sepsis very often have elevated serum lactate, there is a population of critically ill patients with conspicuously normal lactate levels^18^. These critically ill patients with normal serum lactate are poorly characterized in the literature. No explanation exists in the literature to explain this phenomenon of “lactate discordance”. This discordance can potentially result in either over- or under treatment, as physicians sometimes assess resuscitation status through serum lactate levels. For instance, over-resuscitation may ensue from an elevated serum lactate in a patient who is clinically improving, while false reassurance may come from a normal serum lactate in a patient with worsening clinical trajectory^19,20^. The relationship between resuscitation and serum lactate is complex and serum lactate should not be a resuscitation target.

In this study, using three large high-resolution ICU databases (2 from the US and 1 from Spain), we identified the sickest patients who were admitted with sepsis and who had normal serum lactate. We then sought to (1) determine the proportion of the sickest patients with normal serum lactate, and (2) identify features that are correlated with lactate discordance across the 3 datasets.

## Materials and methods

### Data sources

MIMIC-III is a large, open access, single center critical care database containing de-identified data for 61,532 ICU stays admitted at Beth Israel Deaconess Medical Center between June 2001 and October 2012. Version 1.4 of the database is publicly available at https://mimic.mit.edu/.

eICU-CRD is a large, open access, multicenter critical care database holding data associated with 200,859 ICU stays admitted at 58 hospitals across the United States between 2014 and 2015. Version 2.0 of the database is publicly available at https://eicu-crd.mit.edu/. A waiver of consent that has previously been obtained from the Institutional Review Boards of MIT and BIDMC is applicable to these datasets due to their retrospective use of routinely collected EHR data.

ICU23DB is a single center critical care database that contains data for 5617 ICU stays admitted at University Hospital of Tarragona Joan XXIII (HJ23) between 2014 and 2019. HJ23 is a Spanish center with a 30-bed polyvalent ICU. Medical ethical approval of the study protocol was obtained (CEIm-IISPV. Reference: 014/2021).

This study was performed in accordance with the ethical standards as laid down in the 1964 Declaration of Helsinki and its later amendments.

### Study population

The final cohort consisted of ICU patients who were 16 years or older, were in the highest quartile of severity of illness score (SoIS), had sepsis, and had at least one serum lactate measurement recorded during their first day in the ICU. Sepsis was defined as a Sequential Organ Failure Assessment (SOFA) score ≥ 2 plus clinical suspicion of an infection based on initiation of empiric antibiotics and requisition of microbiologic studies^1^. As severity of illness score availability varied for each institution/dataset, different SoIS were used for each dataset, i.e. OASIS^21^ for MIMIC-III, APACHE-IV^22^ for eICU and APACHE-II^23^ for HJ23. However, we believe this does not affect the validity of the findings since all scores have similar discriminative power^21,22^. The data recorded earliest on ICU admission was used to calculate SoIS. None of the scores included serum lactate as a variable. The cohort was restricted to patients with a length of stay in the ICU of at least 24 h. Based on prior literature we defined normal serum lactate as < 2 mmol/L, and high serum lactate as ≥ 4 mmol/L^14,24,25^. The highest serum lactate value within the first 24 h of admission was reported. We used serum lactate levels rather than lactate clearance since it is clinically readily available and has been shown to be more predictive of death^26,27^. Patients with intermediate serum lactate values (2–3.9 mmol/L) were not included in the analysis. A sensitivity analysis was performed on patients who had septic shock.

### Covariates

All Extraction Transform and Load (ETL) processes were carried out using python-pandas (The pandas development team, version 1.10). All queries were implemented and documented in Jupyter notebooks (Project Jupiter, version 6.0.0).

The following variables on admission were extracted from the all the databases: age, sex, serum alanine transaminase, serum aspartate transaminase, blood urea nitrogen, white blood cell count, serum bicarbonate, serum calcium, serum chloride, serum creatinine, serum glucose, platelet count, serum potassium, serum sodium, serum bilirubin, heart rate, mean arterial pressure, and temperature. Furthermore, previous diagnoses were extracted as International Classification of Diseases Codes (ICD) and clustered into related clinical groups relying on the Elixhauser classification^28^.

### Statistical analysis

Statistical analysis was performed in 3 steps: pre-processing, overfitting and generalization: In the pre-processing stage, collinear variables were excluded, numeric values were standardized and missing values imputated. Next, logistic regression (LR)^29^, random forest (RF)^30^, and orthogonal partial least squares discriminant analysis (PLS)^31^ models were built for each data set, cross-validated and performance compared using their accuracy and the Area Under the Receiver Operator Curve (AUROC). During generalization, variables that were only relevant in one data set were removed to limit overfitting and cross-validation and model comparison was repeated. (see Supplementary Methods and Supplementary Fig. S1 for details).

Analyses were carried out in Python version 3.7.3 using pandas^32^. Access to the GitHub repository with the source code is available here: https://github.com/Ps7Pep/LactateDiscordance.

### Sensitivity analysis

We conducted a post-hoc sensitivity analysis for MIMIC-III comparing patients with normal serum lactate levels on day 1&2 with patients with high serum lactate levels on day 1&2. Consequently, we wanted to establish whether lactate discordance on day 1 resulted from the timing of the worst serum lactate, i.e. whether it was drawn before or after resuscitation. By day 2, an elevated serum lactate cannot be ascribed to pre-resuscitation hyperlactatemia. A second sensitivity analysis was limited to patients in septic shock, as defined by the administration of a vasopressor agent (norepinephrine, vasopressin, phenylephrine, epinephrine or dopamine).

### Ethics approval

Medical ethical approval of the study protocol was obtained from the institutions’ IRBs (CEIm-IISPV. Reference: 014/2021, Beth Israel Deaconess Medical Center IRB Protocol #2001P001699) and was granted a waiver of informed consent.

## Results

### Cohort overview

**Figure 1:**
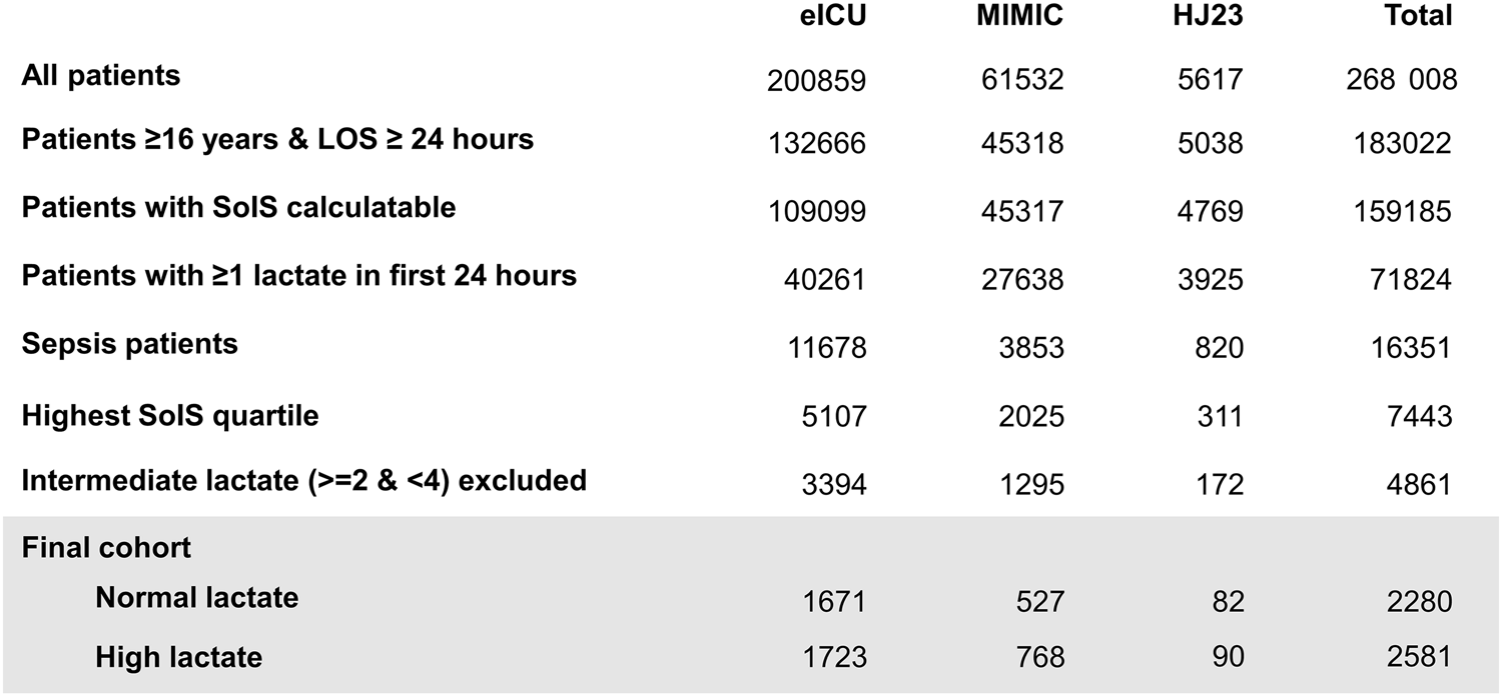
Overview of cohort building and patient numbers by dataset. A total of 16,351 septic patients fulfilling the inclusion criteria were identified. Restricting to highest SoIS quartile and exclusion of intermediate lactate values resulted in a final cohort of 4861 patients. *SoIS* Severity of Illness Score, *LOS* length of stay in the ICU.

A total of 268,008 ICU stays were recorded in the three databases (Fig. 1). After exclusion of patients younger than 16 years and those with a length of stay less than 24 h, 183,022 remained. Of these, all patients that had sufficient information to calculate Severity of Illness Scores (SoIS) at admission and with at least one serum lactate value recorded in the first 24 h of admission were included (71,824). Subsequently, patients with intermediate lactate values ranging from 2–4 mmol/L and those not in the highest SoIS quartile were removed, resulting in a final cohort of 4861. eICU-CRD, MIMIC, and HJ23 contributed 3394, 1295, and 172 patients, respectively. Across all three cohorts, the proportion of patients with normal versus high serum lactate in the highest SoIS quartile was similarly distributed, with normal serum lactate being slightly less frequent (41–49%).

### Patient characteristics

As expected, the final cohort of patients in the highest quartile of SoIS have poor clinical outcomes with 28-day mortality rate ranging from 28 to 38% across datasets (Table 1). ICU length-of-stay was, on average, between 4 and 9 days and similar among the three datasets. Patient characteristics across the three ICU data sets are similar, with similar frequencies of comorbidities, laboratory values, and clinical characteristics. One noteworthy difference is the lower probability of patients in the eICU-CRD to be on mechanical ventilation. Furthermore, mortality rate in the eICU-CRD was also lower than those in MIMIC, yet similar to those in HJ23.

**Table 1:** Overview of baseline characteristics of patients from the three ICU datasets. Overall, characteristics are similar between datasets, with notable exceptions being a higher rate of renal failure, liver disease, and coagulopathy in HJ23. N (%), Median [IQR]. ^a^Medical history as defined by ICD codes.

Dataset	eICU	MIMIC-III	HJ23
Cohort size	3394	1295	172
High lactate	1723 (50.8)	768 (59.3)	90 (52.3)
Normal lactate	1671 (49.2)	527 (40.7)	82 (47.7)
28 days mortality	973 (28.7)	497 (38.4)	49 (28.5)
Length of stay ICU, days	3.7 [2.1, 6.8]	6.7 [3.1, 13.7]	9.3 [4.0, 18.9]
Female	1629 (48.0)	565 (43.6)	68 (39.5)
Age	71 [60, 80]	70 [59, 81]	66 [56, 75]
ALT (U/L)	30 [17, 66]	33 [18, 76]	32 [18, 73]
AST (U/L)	43 [24, 101]	52 [27, 131]	41 [23, 98]
BUN (mg/dL)	38 [24, 57]	36 [23, 55]	41 [27, 57]
WBC (K/mcL)	14.7 [9.1, 21.4]	13.2 [7.9, 20.0]	13.4 [6.4, 21.5]
Bicarbonate (mmol/L)	20 [16, 24]	20 [16, 24]	19 [16, 23]
Chloride (mmol/L)	104.0 [99, 109]	105 [100, 110]	105 [101, 109]
Bicarbonate (mmol/L)	20.0 [16.0, 24.0]	20.0 [16.0, 24.0]	19.4 [15.8, 22.6]
Calcium (mg/dL)	8.1 [7.4, 8.7]	7.9 [7.3, 8.6]	7.9 [7.4, 8.3]
Creatinine (mg/dL)	1.9 [1.2, 3.1]	1.7 [1.1, 2.8]	1.9 [1.3, 3.1]
Glucose (mg/dL)	134 [102, 190]	134 [105, 186]	141 [104, 179]
Platelets (K/mcL)	185 [115, 267]	208 [124, 302]	167 [82, 265]
Potassium (mmol/L)	4.2 [3.7, 4.8]	4.3 [3.8, 4.8]	4.0 [3.5, 4.6]
Sodium (mmol/L)	138 [134, 142]	138 [135, 142]	137 [133, 142]
Bilirubin (mg/dL)	0.8 [0.5, 1.6]	0.8 [0.4, 2.2]	1.0 [0.5, 2.2]
Heart rate	98 [84, 114]	100 [84, 115]	98 [83, 116]
Mean arterial pressure	69 [59, 80]	72 [63, 83]	73 [64, 82]
Temperature	36.8 [35.6, 37.9]	36.7 [35.7, 37.6]	36 [35.5, 36.6]
Peripheral vascular disease^a^	220 (6.5)	124 (9.6)	19 (11.0)
Hypertension^a^	1843 (54.3)	700 (54.1)	78 (45.3)
Hypothyroidism^a^	434 (12.8)	158 (12.2)	12 (7.0)
Diabetes^a^	1201 (35.4)	431 (33.3)	59 (34.3)
Renal failure^a^	521 (15.4)	294 (22.7)	52 (30.2)
Liver disease^a^	286 (8.4)	181 (14.0)	27 (15.7)
Metastatic cancer^a^	167 (4.9)	98 (7.6)	2 (1.2)
Solid cancer^a^	635 (18.7)	63 (4.9)	22 (12.8)
Rheumatoid arthritis^a^	96 (2.8)	50 (3.9)	5 (2.9)
Coagulopathy^a^	405 (11.9)	462 (35.7)	70 (40.7)
Obesity^a^	44 (1.3)	84 (6.5)	20 (11.6)
Deficiency anemia^a^	81 (2.4)	367 (28.3)	32 (18.6)
Chronic pulmonary disease^a^	691 (20.4)	366 (28.3)	28 (16.3)
Chronic neurologic disease^a^	375 (11.0)	200 (15.4)	14 (8.1)
Substance abuse^a^	33 (1.0)	119 (9.2)	21 (12.2)
Heart disease^a^	1376 (40.5)	778 (60.1)	56 (32.6)

### Lactate, SoIS, and mortality

**Figure 2:**
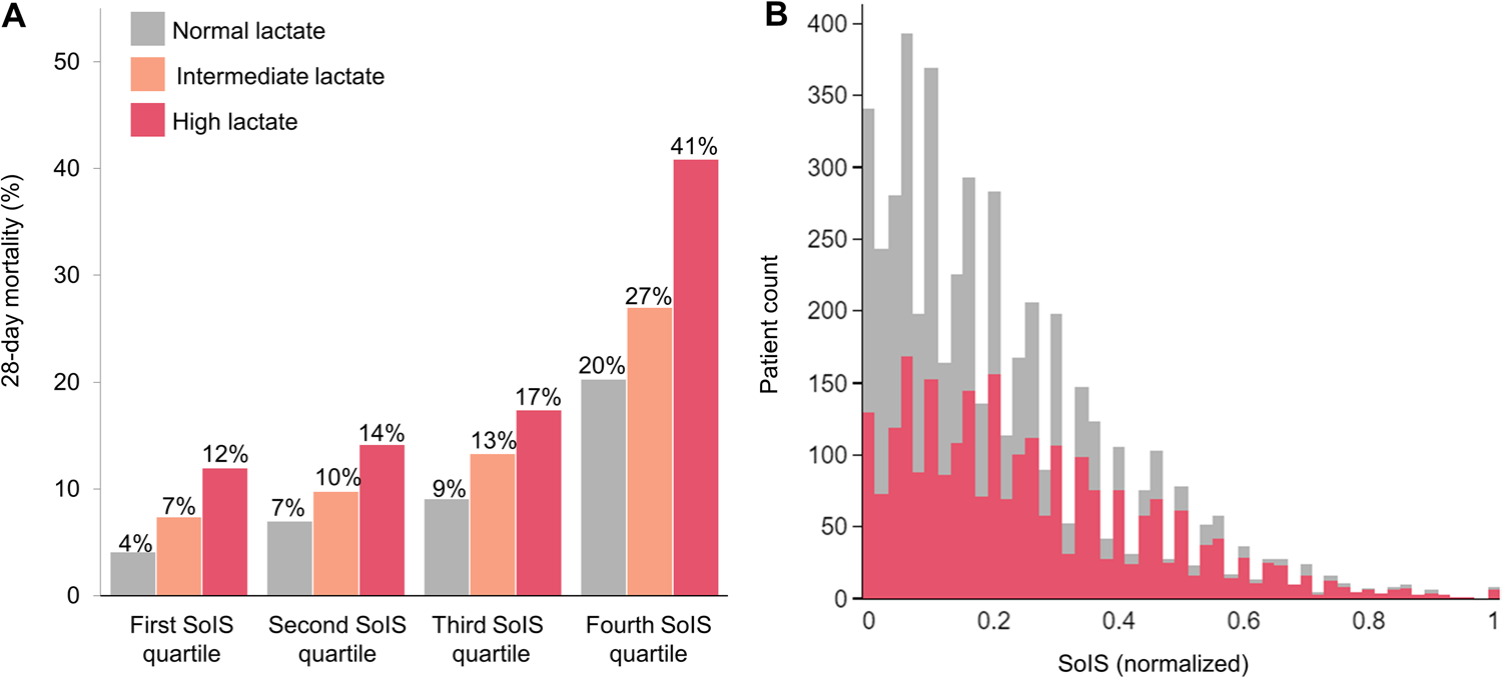
(**A**) 28-day mortality rates by severity of illness score (SoIS) and lactate level. Both higher SoIS and higher serum lactate levels are consistently associated with higher 28-day mortality rates. (**B**) Distribution of SoIS in the normal (gray) and high serum lactate (red) group. Patients with normal serum lactate tend to have lower SoIS. SoIS are normalized to allow comparison across the cohorts.

Lactate levels and SoIS were plotted against mortality rate to demonstrate a positive association between serum lactate and mortality, even within the same SoIS quartile (Fig. 2A). This association was consistent across all quartiles of SoIS. Not surprisingly, plotting of serum lactate levels against normalized SoIS revealed that patients with normal serum lactate tended to have lower SoIS than patients with high serum lactate (Fig. 2B).

### Classification of lactate discordance

We sought to characterize the clinical features associated with normal serum lactate despite high illness severity. To identify these, we used two regression (logistic and partial least square regression) and one machine learning approach (random forests). Across the three datasets, model performance varied by cohort size (Table 2). HJ23 (N = 172) had the highest model accuracy across methods (80–97%), while eICU (N = 3394) had the lowest (77–79%). Within each dataset, model performance was similar for all the methods. Since logistic regression consistently had the highest accuracy and area under the ROC curve, and also due to its interpretability, we will report these results going forward. Details on the hyperparameter tuning can be found in the online supplementary results.

**Table 2:** Comparison of model performance among the three data sets and statistical approaches. HJ23 had the highest model performance, yet lowest cohort size, while the opposite is true for eICU-CRD. For MIMIC and eICU-CRD, model performance is similar across all three methods. *AUROC* area under the receiver operator curve. 95% Confidence Intervals are provided in brackets.

Dataset	Cohort size	Methods	Accuracy	AUROC
eICU	3394	Logistic regression	78.65 (75.56–81.73)	87.36 (84.86–89.86)
		Random forest	77.29 (74.13–80.44)	84.36 (81.65–87.11)
		Partial least square	77.32 (74.17–80.47)	83.70 (80.93–86.48)
MIMIC	1295	Logistic regression	80.31 (75.47–85.15)	87.41 (83.37–91.45)
		Random forest	80.23 (75.38–85.08)	87.11 (83.03–91.19)
		Partial least square	79.54 (74.62–84.45)	87.06 (82.97–91.15)
HJ23	172	Logistic regression	97.14 (91.54–100)	98.69 (94.87–100)
		Random forest	80.00 (66.55–93.45)	89.87 (79.73–100)
		Partial least square	88.57 (77.88–99.27)	97.67 (92.59–100)

### Feature importance

In the logistic regression model, a total of 23 variables were statistically significantly associated with normal serum lactate levels in at least 2 of the 3 databases (see Supplementary Fig. S2). Of these, high levels of serum bicarbonate, serum chloride, history of pulmonary disease, blood urea nitrogen and heart disease were strongest associated with normal serum lactate levels. Conversely, serum sodium, aspartate transaminase levels, history of liver disease, serum glucose concentration and history of heart disease were most positively correlated with high lactate (Table 3). These findings were consistent across datasets, with an exception being the associations of glucose with lactate in the smallest data set HJ23 (OR = 1, N = 172).

**Table 3:**
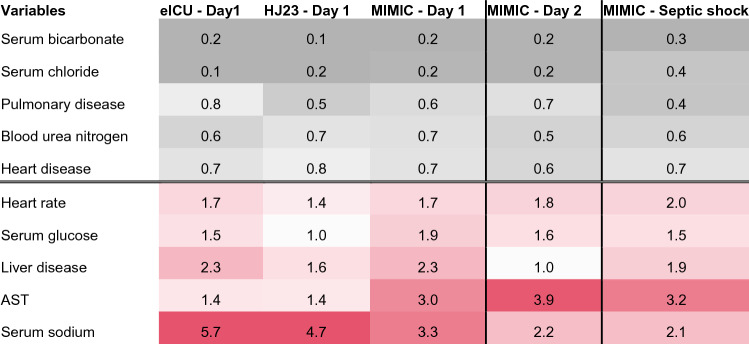
Association of top 10 variables with serum lactate levels in the three datasets (day 1). Second to right column reflects sensitivity analysis for MIMIC-III on day 2. Right column reflects sensitivity analysis limiting analysis to patients with septic shock. An odds ratio (OR) > 1 (red) indicates that variables are associated with high lactate levels. Continuous variables are standardized, i.e., a 1 standard deviation increase is associated with an increase in the shown OR. *AST* Aspartate transaminase.

### Sensitivity analyses

To exclude the possibility that the observed differences in the serum lactate on day 1 were due to the timing of the blood test, i.e. whether it was drawn before or after resuscitation, we repeated the analysis for MIMIC-III at day 2. The day 1 and day 2 models that classified the sickest patients into high vs. normal serum lactate were very similar (Table 3). This argues against the possibility that the model simply grouped the sickest patients to pre- and post-resuscitation states.

Similarly, the subgroup analysis on patients with septic shock, as defined by use of a vasopressor agent, resulted in similar effect size estimates.

## Discussion

We observed that nearly 50% of severely ill septic patients in the ICU have normal serum lactate measurements, which is approximately in line with previous studies^33,34^. The pathobiology of why some severely ill ICU patients have normal serum lactate levels is poorly understood. Here, we identified clinical features that differentiated the sickest patients with normal and high serum lactate at admission. This set of variables was consistent across three large datasets from two countries.

Consistent with past work, serum lactate was found to be an independent predictor of mortality, even within the highest quartile of severity of illness scores. We built both linear models, i.e. logistic regression and partial least square, as well as a non-linear model, i.e. random forest, to identify patient characteristics and biomarkers associated with normal or high serum lactate among the sickest patients. Across the board, these models performed well, as indicated by area under the ROC scores ranging from 0.83 to 0.99 and accuracy scores ranging from 0.77 to 0.97.

The variables most strongly associated with normal serum lactate levels are serum bicarbonate, serum chloride and history of pulmonary disease. Conversely, serum sodium levels, aspartate transaminase and a history of liver disease are associated with high lactate levels. Interestingly, we found heart rate and blood pressure to have a much weaker association with serum lactate levels among the cohort with the highest illness severity scores. Importantly, we found the model on day 2 post-admission to discriminate between normal and high serum lactate levels equally well as the model on day 1. This observation makes it unlikely that the observed differences are solely due to the timing of the serum lactate determination, i.e. pre- or post-resuscitation.

Past work has consistently demonstrated that elevated serum lactate is associated with increased ICU and hospital mortality^14,15,18^. These findings have contributed to management strategies driven by serum lactate level, with guidelines recommending early measurement^35^ and some trials demonstrating clinical benefit to a serum lactate-targeted approach^36^. However, despite decades of work to elucidate and reframe the role of lactate in health and illness^37^, hyperlactatemia is conventionally equated with “hypoperfusion” in many clinical settings^38^, a potentially harmful oversimplification. When comparing our results with the recently established sepsis phenotypes^16^, the high serum lactate groups seems to most closely resemble the delta phenotype, given high serum lactate values, a high mortality rate, elevated AST and low bicarbonate. Contrarily, the delta phenotype was not associated with high serum sodium and low serum chloride, as it was the case with our high serum lactate group. Since this study compares patients at admission, it does not cover interventions and therefore may differ from the results of Seymour et al.^16^.

Increased production of lactate in critical illness has been attributed to reduced oxygen delivery to or utilization by tissues, with lactate as the “waste product” of lactatogenic glycolysis^38^. However, derangements in the delivery and utilization of oxygen do not completely explain lactate production, nor does an understanding of a single tissue bed reflect an entire organism, or a syndrome as protean as sepsis—a dysregulated, catabolic state. Infection and increased circulating catecholamines, both found in sepsis, are independently sufficient to induce lactate production. Investigators have demonstrated that glycolysis proceeds to lactate production under aerobic conditions^39^ and there is growing interest in lactate’s role as an energy source, gluconeogenic precursor, signaling molecule, and, altogether, adaptive response to stress or illness^37,40^.

The data presented here are observational. It is impossible to disentangle whether an observation leads to, results from, or simply co-occurs with an elevated serum lactate. Serum bicarbonate is correlated with serum lactate, and in turn influences serum chloride to maintain electrical neutrality, but it is unclear why they are associated with normal rather than elevated serum lactate. Liver disease (and its associated coagulopathy) is unsurprisingly more likely among those with high serum lactate, but it is interesting that AST, but not ALT, correlates with serum lactate among the sickest ICU patients. Even more interesting are the findings that abnormal BUN, serum creatinine and platelet count are associated with a normal serum lactate in this cohort. While speculative, a possible explanation could be that renal compensation tends to be slow and therefore renal disfunction is not associated with high serum lactate at baseline. The observed lack of a significant association between history of hypertension and mean arterial blood pressure fits the current understanding that lactate is not a marker of tissue hypoxia in sepsis^37,40^. However, it is possible that observed biomarker trends are as much a consequence of epiphenomena or unmeasured confounding as they are reflective of metabolic flux.

Key strengths of this study are the use of large datasets from two countries. The similarity of the findings across the three databases is striking, however, it does not guarantee generalizability of the observations especially to ICUs that may differ in their patient demographics and practice patterns. Furthermore, consistency of findings on day 1 and day 2 after admission, as well as for patients with septic shock, is encouraging.

We hope the analysis presented here inspires further studies to better understand this phenomenon of lactate discordance among ICU patients with the highest acuity. In addition, we hope that it also stimulates novel research that would bridge the gap between model organism studies and small flux analyses with large, high-resolution data to generate hypotheses for the role of and reason for lactate production in critically ill patients. With growing recognition that lactatemia is much more than a monolithic marker of tissue perfusion, these data serve as a platform to deepen and diversify our understanding of the role of lactate in sepsis. Given the significant overlap of the high serum lactate group with the delta phenotype, this study could provide new thought starters to further develop the sepsis phenotypes described by Seymour et al.^16^.

In conclusion, we established high performing statistical models that consistently identify features associated with normal serum lactate levels in critically ill patients with sepsis across three international datasets. These patient characteristics and clinical parameters may serve as a starting point for future studies to better understand the underlying pathophysiological mechanisms of lactatemia and derive clinical implications for critically ill patients with normal lactate levels.

## Supplementary Information

Supplementary Information.

## Data Availability

MIMIC-III and eICU-CRD are publicly available from Physionet [https://www.physionet.org/about/database]. No de-identified, anonymized version of ICU23DB is available as of this date—but can be made available from the corresponding author on reasonable request.
