# Supplementary material for: Understanding critically ill sepsis patients with normal serum lactate levels: results from U.S. and European ICU cohorts
Source: Sci Rep. 2021 Oct 8;11:20076. doi: 10.1038/s41598-021-99581-6 (PMC8501011; doi:10.1038/s41598-021-99581-6)
Supplement: Supplementary file 1 — Supplementary Information. [file 41598_2021_99581_MOESM1_ESM.docx]

**Understanding critically ill sepsis patients with normal serum lactate levels – results from U.S. and European ICU cohorts**

Christopher M. Sauer MD MPH^1,2&^, Josep Gómez PhD^3,4&*^, Manuel Ruiz Botella BSc^3,4^, David R. Ziehr MD^5,6^; William M. Oldham MD PhD^6,7^, Giovana Gavidia PhD^8^, Alejandro Rodríguez MD PhD^3,4^; Paul Elbers MD PhD^2^, Armand Girbes MD PhD^2^, Maria Bodi MD PhD^3,4^, Leo Anthony Celi MSc MD MPH^1, 9,10^

^&^Shared first authorship

Methods

**Model building process**

In the pre-processing stage, collinear variables and those with more than 50% of missing values, were excluded, while for the rest, missing values were imputed using the Multivariate Imputation Chained Equations (MICE) method (37). Finally, numeric values were standardized by subtracting the mean value and dividing by the standard deviation.

In the overfitting stage, logistic regression (LR) (25), random forest (RF) (26), and orthogonal partial least squares discriminant analysis (PLS) (27) were applied to each database separately to obtain a total of 9 distinct models. Next, a 5-fold cross-validation forward feature selection was applied to each model, and the set of variables that obtained the highest area under the receiver operator characteristic (AUROC) curve or AUC were selected for step two. Consequently, each algorithm was re-launched and supplied with the set of variables obtained in the previous step, using again a 5-fold cross-validation strategy. The model that obtained the best accuracy was selected to study the variable's importance using the regression coefficient for LR and PLS, and the Gini index (38) for RF.

In the generalization stage, the variable sets obtained for each database were unified in a unique variable set containing only the variables that were found to be important in at least 2 of the 3 databases. Thereby, variables that contributed to overfitting of the models were excluded, keeping those which were selected across the three databases. Finally, each algorithm was re-launched using 5-fold cross-validation for each database but supplied with the global set of variables (selected by the models across the 3 datasets), and the model that obtained the best accuracy was selected to study the final models and their variable importance panel.

**Hyperparameter tuning**

For logistic regression model, the only hyperparameter we tested was class balancing from the Python package sklearn. However, as classes were only slightly unbalanced (51/49 in eICU, 59/41 in MIMIC, 52/48 in HJ23) the results did not significantly improve (see suppl. table S1), so we maintained the model without class balancing.

For the random forest models, hyperparameter tuning focused on finding the optimum number of trees and their optimum depth. After testing several options (see table S2), the final parameters were set as 200 trees with depth 10, as no significant improvements justified increasing the analysis resources.For the partial least squares regression models we studied the number of components and the standardizing of the variables in the OPLS model. After making a grid for hyperparameter tuning, we found that there were no significant improvements that justify more than 3 components or standardizing the data (see table S3).

Supplementary figures

**Supplementary Figure S1** Overview of the model building process: Output of the first models were combined during cross-methods feature condensation to exclude variables that were only selected in one of the datasets. 5-fold cross-validation resulted in the final model output. LR: Logistic regression, RF: Random Forests, PLS: orthogonal partial least squares discriminant analysis


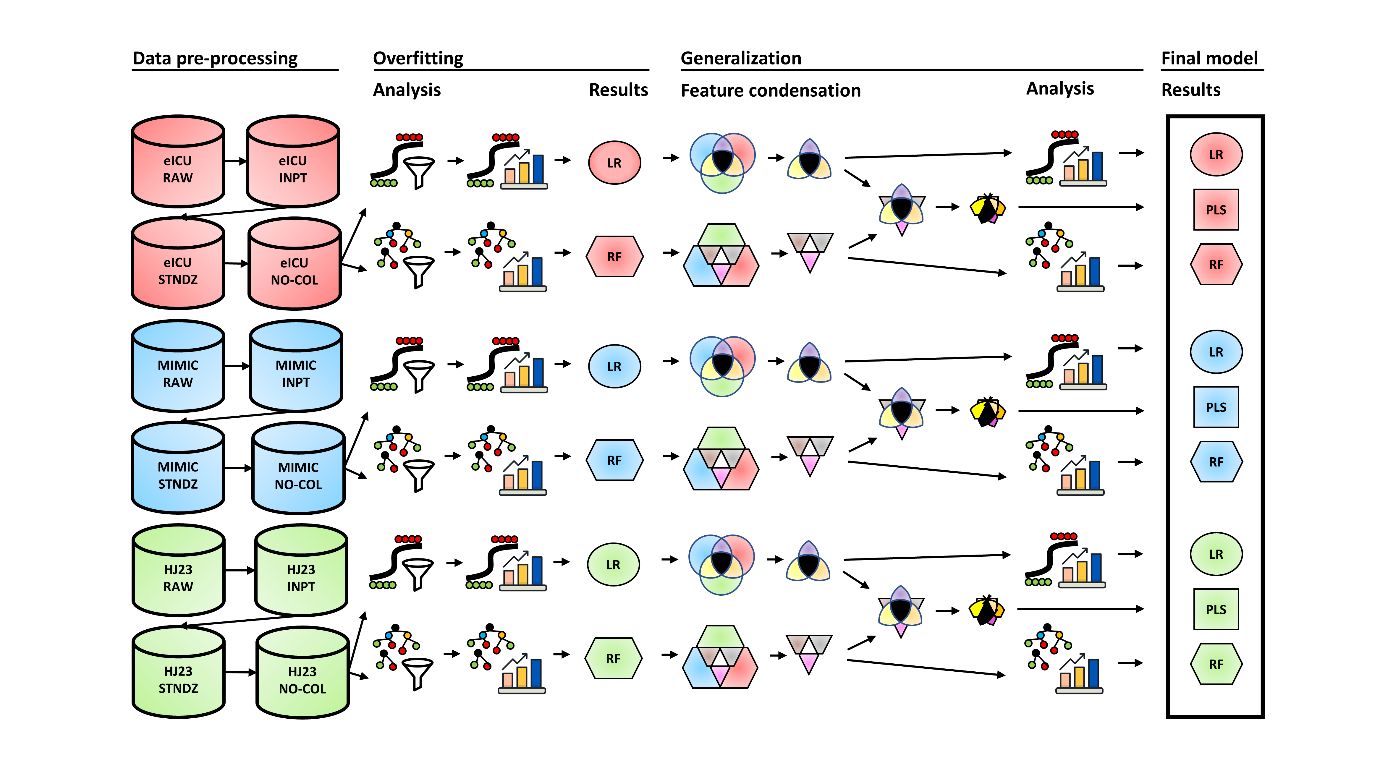


**Supplementary Figure S2** Association of all variables with serum lactate levels in the three datasets. An odds ratio (OR) >1 indicates that variables are associated with high lactate levels. Visualization on a logarithmic scale. Continuous variables are standardized, i.e. a 1 standard deviation increase is associated with the plotted OR. *Categorical variable based on ICD registration. BUN: Blood urea nitrogen; AST: Aspartate transaminase


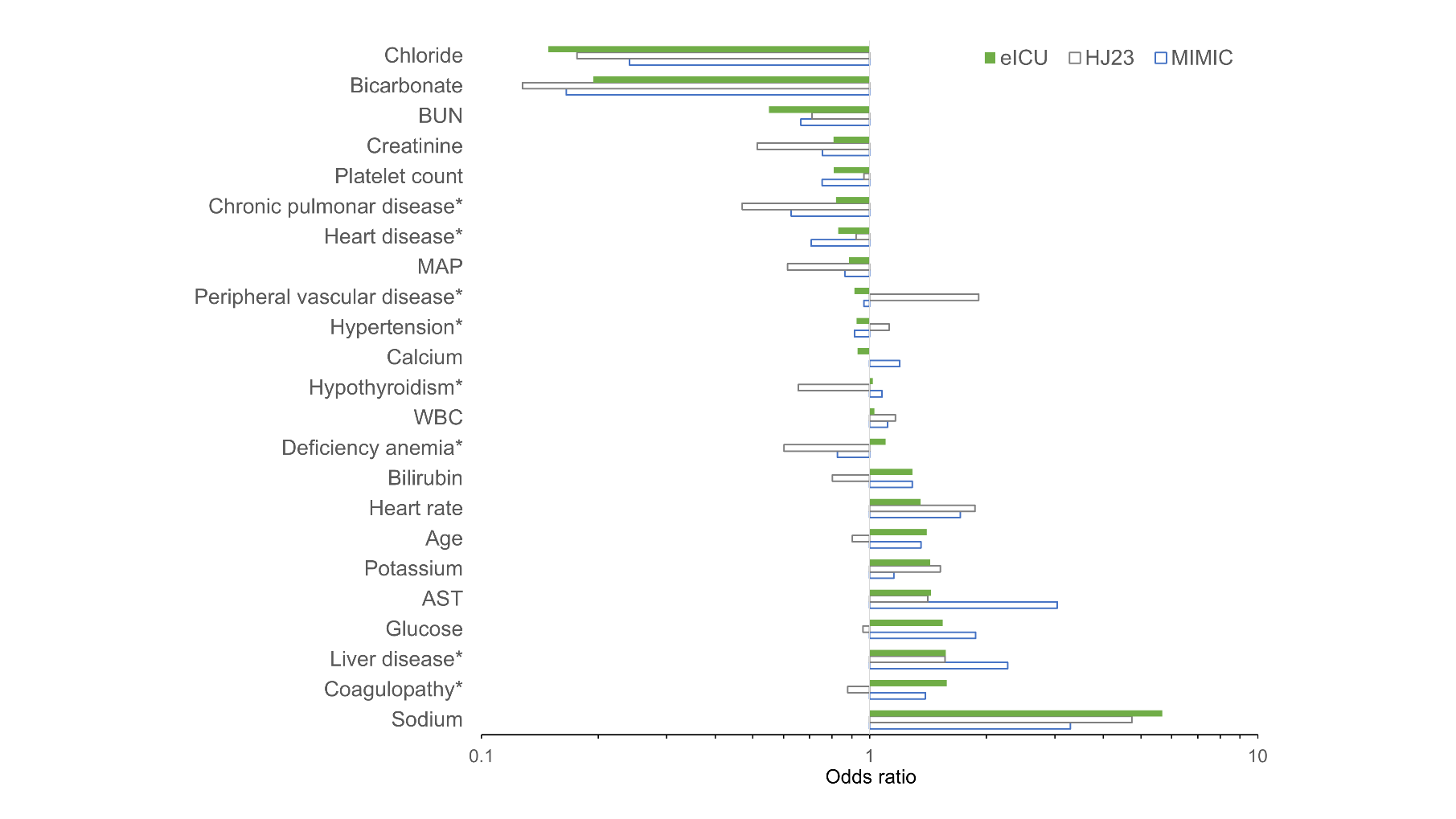


Supplementary tables

**Supplementary Table S1** Comparison between default and balanced logistic regression

| **Dataset** | **Accuracy** | **Accuracy balanced** | **AUROC** | **AUROC balanced** |
| --- | --- | --- | --- | --- |
| eICU | 78.65 (75.56 – 81.73) | 78.94 (75.87 – 82.00) | 87.36 (84.86 – 89.86) | \| 87.36 (84.86 – 89.86) \| \| --- \| |
| MIMIC | 80.31 (75.47 – 85.15) | 79.84 (74.96 – 84.73) | 87.41 (83.37 – 91.45) | 88.26 (84.34 – 92.18) |
| HJ23 | 97.14 (91.54 – 100) | 97.14 (91.54 – 100) | 98.69 (94.87 – 100) | 98.69 (94.87 – 100) |

**Supplementary Table S2** Random Forest classification performance (AUROC) at different number of trees and depth.

| **Hyperparameters** | | **eICU** | **MIMIC-III** | **HJ23** |
| --- | --- | --- | --- | --- |
| **Number of trees** | **Depth** |  |  |  |
| 50 | 10 | 84.25 | 86.36 | 90.20 |
| 100 | 10 | 84.36 | 86.01 | 88.56 |
| 200 | 5 | 82.60 | 84.25 | 89.22 |
| 200 | 10 | 84.36 | 87.11 | 89.87 |
| 200 | 20 | 84.19 | 86.82 | 89.22 |
| 500 | 10 | 84.45 | 87.32 | 89.21 |

**Supplementary Table S3** Partial Least Squares classification performance (AUROC) for different number of components with and without standardization.

| **Hyperparameters** | | **eICU** | **MIMIC-III** | **HJ23** |
| --- | --- | --- | --- | --- |
| **Number of components** | **Standardization** |  |  |  |
| 1 | No | 79.58 | 84.52 | 94.39 |
| 3 | No | 83.70 | 87.06 | 97.67 |
| 5 | No | 84.08 | 86.34 | 97.00 |
| 10 | No | 83.92 | 86.64 | 96.49 |
| 1 | Yes | 79.87 | 84.52 | 92.98 |
| 3 | Yes | 83.53 | 87.04 | 98.00 |
| 5 | Yes | 83.95 | 86.79 | 96.49 |
| 10 | Yes | 83.92 | 86.64 | 96.49 |
